# Are they really refusing to travel? A qualitative study of prehospital records

**DOI:** 10.1186/1471-227X-6-8

**Published:** 2006-09-19

**Authors:** Deborah Shaw, Jane V Dyas, Jo Middlemass, Anne Spaight, Maureen Briggs, Sarah Christopher, A Niroshan Siriwardena

**Affiliations:** 1 East Midlands Ambulance Service NHS Trust, Ambulance Training Centre, 4 Proctors Road, Outer Circle Road, Lincoln, UK; 2 Trent Research and Development Support Unit, Division of Primary Care, 13th Floor, Tower Building, University of Nottingham, UK; 3 Nottingham Primary Care Research Partnership, Hucknall Health Centre, Curtis Street, Hucknall, Nottingham, UK; 4 University of Lincoln, School of Health and Social Care, University of Lincoln Court 11, Apartment 1, Room 2, Campus Way, Lincoln LN6 7BG, UK

## Abstract

**Background:**

Refusal by the patient to travel after calling an emergency ambulance may lead to a preventable waste of scarce resources if it can be shown that an alternative more appropriate response could be employed. A greater understanding is required of the reasons behind 'refusal to travel' (RTT) in order to find appropriate solutions to address this issue. We sought to investigate the reasons why patients refuse to travel following emergency call-out in a rural county.

**Methods:**

Written records made by ambulance crews for patients (n = 397) who were not transported to hospital following an emergency call-out during October 2004 were retrospectively analysed.

**Results:**

Twelve main themes emerged for RTT which included non injury or minor injury, falls and recovery after treatment on scene; other themes included alternative supervision, follow-up and treatment arrangements or patients arranging their own transport. Importantly, only 8% of the sample was recorded by ambulance crews as truly refusing to travel against advice.

**Conclusion:**

A system that facilitates standardised recording of RTT information including social reasons for non-transportation needs to be designed. 'Refused to travel' disclaimers need to reflect instances when crew and patient are satisfied that not going to hospital is the right outcome. These recommendations should be considered within the context of the plans for widening the role of ambulance services.

## Background

Ambulance services in the United Kingdom (UK) are legally required to attend emergency '999' and general practitioner (GP) calls unless valid treat-and-leave or despatch triage protocols are in place. Previous literature from the UK and North America sometimes refers to 'inappropriate calls'[1,2] which it is argued could lead to fewer resources being available to respond to life-threatening incidents. The potential problem is even greater in large rural counties where long distances are travelled.

There is considerable overlap between the concept of an 'inappropriate call', non-transport (or non-conveyed call) and 'refusal to travel' (RTT). The Department of Health cites the national average of non-conveyed calls as 17% [3]. Marks et al. in a study in the East Midlands (UK) identified that over one third of these were due to falls in elderly people; patients who were categorised as 'refusal to travel' (RTT) formed a further subset (up to half) of those not transported [4]. Patients or relatives may genuinely request transport but they may also agree to sign a non-transport disclaimer after informal advice from an ambulance crew that transport may be unnecessary; another factor raised but not elucidated in previous research [5].

Alternatives to ambulance attendance may provide a possible solution[6] although studies in North America have found that, of those who refuse to travel, almost half require further attention within one week,[7] a significant minority require hospital admission,[8] and many agree to be admitted following telephone advice from medical personnel [9,10].

Recent research from the UK has shown that prior telephone assessment of non-urgent calls by nurses or paramedics may identify patients less likely to need emergency department care [11]. Possible methods to address RTT include alternative community services, alternative transport,[12] and specific interventions such as community falls programmes.

In order to inform the development of such interventions a greater depth of understanding is required of the reasons for RTT. This study sought, using mixed methods, to investigate why patients refuse to travel following emergency call-out in a rural county.

## Methods

This study was conducted during 2003 and 2004 in Lincolnshire, a large rural county for which East Midlands Ambulance (formerly Lincolnshire Ambulance and Health Transport) NHS Trust provides prehospital services to a population of over 700,000. At the time of the study all patients requesting an emergency ambulance were assessed by nonmedical trained dispatchers using a decision support tool, the Advanced Medical Priority Dispatch System (AMPDS), and if categorised as needing an ambulance response were required to be transported to hospital unless they completed a 'patient not treated/transported' form, known colloquially as a 'refusal to travel' form. No medical or nurse assessment was used to assess or prioritise calls at the time of the study.

Free text data were collected retrospectively from standard 'patient not treated/transported' forms written by paramedic or ambulance technician crews (clinicians) and signed by patients to confirm that they received advice and declined transport. Corresponding patient record or report forms, also written by clinicians, were also used to obtain information on patient details, presenting complaint, observations made and treatments administered. It was the responsibility of the crew to ensure where possible that the record was completed in cases where transport was declined. This study occurred before protocols were in place to enable crews to 'see and leave' or 'see and treat' patients. Numbers of forms completed were recorded and data on travelling times involved were obtained from the Computer Aided Despatch (CAD) system. Costs were derived from trust financial data.

### Ethical approval

Ethical approval for this study was obtained from Lincolnshire Local Research Ethics Committee (reference no. 04/Q2405/46).

### Sample selection

There were 76635 emergency requests resulting in activation of an ambulance response from April 2003 to March 2004. This resulted in 63650 transportations, 9068 instances of refusal to travel (RTT), 967 deaths and 2950 calls with other reasons for non-transportation (see below). The rate of RTT as a proportion of the total requests was therefore 11.8% and this was stable throughout the year without significant seasonal variation. A single month (October 2004) was therefore selected as typical for both the pilot and the main study. Consecutive cases of RTT were selected for analysis until saturation was reached. Cases where treatment only had been declined but transport accepted were excluded.

### Data collection and analysis

An initial pilot study (n = 50) was undertaken to develop the data collection tool and template for the full study. 397 (i.e. a further 347) records were required to achieve theoretical saturation, i.e. no new data emerging to contribute to theoretical development of new or existing categories. Free text information from records was transcribed using data collection headings agreed by all the authors (Table 1). Recorded data were entered into QSRN6 for analysis [13] Data were further organised into categories and grouped into themes by two independent researchers (JM, DS). These were described and named after discussion by the whole research team (which included paramedic, nursing, medical, allied health professional and lay members) reflecting a multidisciplinary perspective. Descriptive statistics were used to assess frequencies within themes.

**Table 1:** Data collection headings used to organise data from the AS34 (RTT form) and AS9 (patient record) developed in the pilot study

**Data collection Heading**	**Source and content of information**
Date of Incident and job number	Unique identifier for every emergency call. Patient details were omitted from the research data but the unique identifier ensured that the researchers were able to trace the original records.
Vehicle call sign	'Cross check' to ensure that in cases which involved multiple patients under the same identifier, for instance in the case of a road traffic collision, or where a patient name had not been recorded or in cases where the scanning system used to store information onto the record database misread job numbers leading to two records with the same identifier being stored, the correct record could be identified.
Presenting condition	The presenting condition was the information recorded in the 'chief complaint' box (on AS9)
Immediate treatment given	Any treatment that was recorded on the AS9 as being provided to the patient by the ambulance clinician on scene.
Refusal against advice	Instances where the clinician has explicitly noted on the AS 34 or AS 9 that the patient had 'refused to travel' against advice.
Clinical reason for non-transportation where stated	Where the reason, as recorded by the clinician for non-transportation, suggested that the injury or condition did not require hospital treatment/there was no injury. This information was gathered from the reasons recorded on the AS34 and/or the free text history box on the AS9.
Patient reason for refusal where stated	This heading was used to note the reasons for non-transportation where they were explicitly recorded as originating from the patient. This information was gathered from the AS 34 and/or the AS 9.

A count of clinical observations recorded on the patient report was included in the analysis because this indicated that the crew had made a clinical assessment of the patient. This was important because some instances of RTT, for example when the patient was intoxicated, could involve refusal of clinical assessment as well as transport.

## Results

An audit during April 2003 to March 2004 identified 9068 instances of RTT out of 76635 calls comprising 11.8% of emergency calls throughout the year in Lincolnshire. There were also 2950 cases where a patient did not travel where death was confirmed on scene by a doctor, the patient refused to provide details or to sign a disclaimer, or alternative means of transportation occurred, for example by air ambulance. The overall rate of non-transportation for the year was therefore 16.9%. RTT was estimated to cost £1.45 million. This was calculated from an average cost per emergency activation of £160.02 (a figure derived from trust data), 9068 episodes of RTT × £160.02 per activation costing £1.45 million for the year. This assumed that RTT activations were a similar cost per activation as non-RTT emergency calls.

### Presenting condition

Presenting conditions were recorded and grouped into six main problem themes (Table 2). In some instances there was more than one presenting condition recorded for a single patient. Sometimes information was recorded as a medical diagnosis such as chronic obstructive pulmonary disease (COPD) whilst at other times using lay terminology, such as breathing difficulties. Whatever the preferred terminology failure to transport patients to hospital was usually explained in medical terms. Most patients who 'refused to travel' were recorded as having a medical condition or problems with mobility. Themes were further subdivided into specific or more general conditions.

**Table 2:** Presenting themes, conditions and prevalence

**Themes**	**Number of presenting conditions (n = 409)**
**Medical (153 [37.4%])**	
Named Conditions (includes):	112
*Respiratory (inc breathing, hyperventilation)*	41
*Hypoglycaemia*	25
*Chest pain*	15
*Fit*	10
*Abdominal pain*	9
*Medication problem*	4
*Muscular*	3
*Other (Including pregnancy)*	3
*Sinus problems*	1
*Neurological*	1
Collapse	31
Non-specifically unwell	10
**Mobility (134 [32.8%])**	
Falls	131
General difficulty with mobility	3
**Actual/potential trauma (87 [21.3%])**	
RTA	47
Minor Injury – non-fall	23
Assault	13
Fire call	2
Entrapment – non-RTA	1
Ingested foreign object	1
**Toxic (27 [6.6%])**	
**Psychological (6 [1.5%])**	
**Social (2 [0.5%])**	

### Clinical observations

Clinical observations made by ambulance staff were routinely recorded in 229 of 397 (57.7%) cases. Electrocardiography (ECG) was included as a form of immediate management because it may have indicated that the patient's condition was potentially serious.

### Immediate management

Over two thirds (69.8%) of patients in the sample who 'refused to travel' received immediate management at the scene (Table 3). The commonest category of immediate management where patients subsequently refused to travel were those cases where the patient was assisted off the floor, out of the bath or elsewhere, either to bed or to a suitable chair.

**Table 3:** Type of immediate management given to patients

**Immediate management themes derived from the analysis of the records (n = 397)**	**Categories**
**Investigations 6 (1.5%)**	*ECG*
**Treatment 75 (18.9%)**	*Glucose given (*22 [5.5%])*
	*First aid dressing (*20 [5.0%])
	*Ankle/wrist strapped*
	*Nebuliser*
	*Oxygen*
	*Airways cleared*
	*Narcan*
	*Aspirin*
	*GTN*
**Other management 87 (21.9%)**	*Lift from floor/bath etc (*60 [15.1%])
	*Advised/observed taking medication*
	*Delivery of baby*
	*Catheter bag replaced*
	*Management prior to arrival by others at scene*
	*Reassurance (hyperventilation)*
	*Paper bag*
	*Hand/arm released*
**Referral 2 (0.5%)**	*Out of hours doctor called*
**Transport 5 (1.3%)**	*Transport other than to hospital*
**Refused treatment 14 (3.5%)**	

*All other categories less than 5%

Immediate management was sometimes administered by others at the scene rather than the ambulance crew, for example one patient received oxygen from the fire service. Giving oral glucose to patients suffering hypoglycaemia resulted in clinical improvement obviating the need for transport. The minor theme of 'transport' reflected cases where transport was provided other than to hospital, for example, to a patient's home when there had been a fall in a public place. A small number of patients, particularly cases associated with alcohol consumption or assault, refused treatment as well as transport to hospital. Clinicians had no choice but to make a decision about the patients' competence to refuse treatment in this situation.

### Reasons for 'refusal to travel'

There was a clear statement of why a patient 'refused to travel' in 80.6% (320/397) of cases; over half of these 68.8% (220/320) reported the clinician viewpoint, 31.3% (100/320) of the statements reported reasons that the patients themselves had given. The remaining forms gave no explanation other than the patient refused. Where the reason was recorded, there were seven themes under reasons given by patients (Table 4) and four themes for reasons given by the clinicians (Table 5).

**Table 4:** Patients' stated reasons for refusing to travel

Reasons given by patients but recorded by crew Themes * (n = 100)	**Quotations recorded on the AS34**
**Patient instigated follow-up 52 (52%)**	*'Patient preferred to travel to hospital with husband in car'* *'Patient to take self to A&E if pains worsens'*
	*'Patient prefers to see own GP tomorrow'* *'Felt isolated episode and did not wish to travel to hospital having recovered'*
	*'Patient's parent will keep an eye and call back if needed'* *'Will call again if side effects later'*
**Alternative supervision arranged 19 (19%)**	*'Left in police custody'* *'Patient left with care staff'* *'Patient put in contact by phone with YMCA outreacher'*
**Follow-up in place 3 (3%)**	*'Had bloods taken at hospital in morning re investigation into faints. Did not wish to attend A&E'*
	*'Patient due on ward for knee operation'*
**Terminal illness 2 (2%)**	*'Terminal, wish to remain at home'* *'Patient family requested to keep at home. District nurse on scene'*
**Ambulance used as alternative management 5 (5%)**	*'Off feet. Family friend rang GP, wouldn't visit told to ring 999'*
	*'Patient called ambulance just to have old wound dressed'*
**Social 7 (7%)**	*'Patient said she had been drinking most of the day and was going to walk home'* *Patient threatening to 'have crew for assault if touched or forced to go to hospital'*
	*'Did not want to leave disabled daughter'* *Doesn't like hospital and has to look after wife'*
**Other 12 (12%)**	*'Didn't know why ambulance had been called, became agitated and aggressive'* *'Neighbour rang against wishes of the patient'*.
	*'Does not travel well, going home with partner'*

**Table 5:** Clinicians' stated reasons for patients 'refusing to travel'

**Reasons given by clinicians/crew Themes (n = 220)**	**Quotations recorded on the AS34**
**Non injury/minor injury 120 (54.5%)**	*'Fall – no injury'* *'Minor injuries only'* *'RTA – no injuries'*
**Patient responded to treatment/recovered/significantly improved 54 (24.5%)**	*'Condition improved after treatment'* *'Baby delivered, no need for transportation'*
**For assessment by other clinician (usually doctor) 2 (0.9%)**	*'Examined by out of hours doctor'*
**No medical emergency/social problem/inappropriate call out 12 (5.5%)**	*'No medical emergency (4*^*th*^*visit in last 20 hours)'* *'Faint – couldn't get through to Out of hours [OOH] doctor so called ambulance'* *'Not appropriate as social problem'* *'No reason for call-out'* *'Can't cope – wants to go but only if wife can go as well'*
**Explicitly recorded refused to travel against advice 32 (14.5%)**	*'Refused against strongest advice and concerns'* *'Refused transport four times'* *'Refused – pain in lumbar region following RTA'* *'Refused – liased with out of hours GP'*

*The themes and categories were identified from the data and agreed by the research group

In just over half of the cases where there was a clear statement of why a patient 'refused to travel' (54.5%; see Table 5), there was either minor injury or no apparent injury. In a further quarter (24.5%) of cases the patient responded well to treatment. In 5.5% of cases the clinician recorded that there was no medical emergency and labelled this as a social problem or inappropriate call-out.

However, in only 32 of 397 (8.0%) cases did the ambulance crew explicitly record that the patient refused to travel against advice.

## Discussion and conclusion

### Summary of main findings

This study has shown that the term 'refusal to travel' was misleading as only 8% of our sample were recorded as not travelling to hospital against professional advice. The findings show that recorded reasons for completing a 'refusal to travel' form were complex and diverse. Sometimes these consisted of patient generated reasons and at other times clinically derived reasons. These were expressed variably as medical diagnoses or using lay terminology and in physical terms more often than psychological or social descriptions. In most (over 90%) cases, although a RTT form was completed, it appeared that refusal to travel was a negotiated shared decision. Although RTT was estimated to cost £1.45 million this was not an entirely wasted resource because ambulance crews did provide an on-site assessment. However, it is clear from the data that alternatives to an ambulance response based on prior telephone assessment may have been a more appropriate use of some of this resource.

### Comparison with existing literature and implications for future practice

One third of patients who subsequently 'refused to travel' had suffered trips, slips and falls without sustaining injury, a finding consistent with other studies [4] Management for these patients, often the frail elderly, was to lift them from the floor or bath. Telephone triage before sending an ambulance may have determined an alternative response, reducing the likelihood of transfer to hospital [11] Although meeting NHS needs, little is known of the acceptability of this approach to patient or carer and whether other psychological or environmental needs are being met.

Third-party requests against the patient's wishes resulting in RTT may have been prevented by a question incorporated into the triage process, for example "does the patient know about the call-out being made on their behalf?" If the answer is "no", an attempt could be made, for example using a triage nurse, to speak with and gather information directly from the patient, thereby potentially facilitating a more appropriate response. Obviously where the patient is unconscious or involved in a road traffic accident this is not a consideration.

Calls were labelled by clinicians as inappropriate for very few patients. Where calls were for trivial complaints or by frequent callers, mental health issues may have been an underlying factor. Calls requesting medication supplies could have been addressed by patient education.

### Strengths and limitations of the study

One limitation of the study included the data source, the written clinical records, from which qualitative information which was derived. The data gleaned from these records included reasons given by patients but interpreted and documented by crews. These were sometimes brief, consisting of a few words and therefore not enough to gain any in-depth understanding of why certain phenomena were occurring. It cannot always be assumed that the interpretations of patient reasons were accurate.

The focus on physical rather than psychological or social terms by clinicians could have been due to the way the form was constructed or because of expectations of clinicians. For example, only one social problem was explicitly recorded in our sample.

Another anomaly in the recording system occurred when patients had recovered significantly since the call, which obviated the need for transport. The system at the time of the study prevented ambulance crews from doing anything other than take patients to hospital, even if their condition did not warrant it, unless a 'refusal to travel' form was signed. Not transporting after treatment of hypoglycaemia, for example, has been shown to be safe [14] with adequate follow up[15] but was not an option for crews at the time of the study. A 'treat and leave' protocol would have removed the perception that these successfully treated cases were inappropriate calls instead of appropriate requests with satisfactory outcome for patient and crew.

The strength of the study is in its careful analysis of qualitative data from a multidisciplinary including patient perspective.

### Conclusions and suggestions for future education and research

New systems for responding to emergency calls, whether in the UK or in comparable health systems abroad, including the use of telephone triage, on-scene assessment and referral could lead to changes in the rate of 'refusal to travel'.

The data suggested that the processes leading to refusal to travel and non-transportation to hospital were complex. Many interacting factors were often involved, including medical, emotional and social. In-depth interviews with ambulance crews and patients would enable a fuller understanding of these factors.

There are implications for future ambulance service education and training, not just in recording information, but also shared decision making, informed consent and greater integration with other health professionals in order to develop more multi-disciplinary solutions for those cases not leading to transport to hospital.

Further research is recommended into the impact of Emergency Care Practitioners and Community Paramedics on non-transportation. These recommendations should be considered within the context of the new Government plans for widening the role of ambulance services outlined in the Bradley report [16].

## Competing interests

Jane Dyas is employed by Trent RDSU and mentor to the project, which was funded by a capacity building grant. She was, however, not an applicant for the funding.

## Authors' contributions

All authors participated in the conception and design of the study, and the analysis of data. JM and DS participated in the acquisition of data. DS, NS and JD drafted and revised the manuscript. DS, JD, AS, MB, SC and NS read and approved the final manuscript.

## Pre-publication history

The pre-publication history for this paper can be accessed here:


